# Homoharringtonine targets Smad3 and TGF-β pathway to inhibit the proliferation of acute myeloid leukemia cells

**DOI:** 10.18632/oncotarget.16956

**Published:** 2017-04-08

**Authors:** Jian Chen, Qitian Mu, Xia Li, Xiufeng Yin, Mengxia Yu, Jing Jin, Chenying Li, Yile Zhou, Jiani Zhou, Shanshan Suo, Demin Lu, Jie Jin

**Affiliations:** ^1^ Department of Hematology, The First Affiliated Hospital, Zhejiang University School of Medicine, Hangzhou, China; ^2^ Institute of Hematology, Zhejiang University, Hangzhou, China; ^3^ Laboratory of Stem Cell Transplantation, Ningbo First Hospital, Zhejiang, China; ^4^ Hematology Department of Ningbo Medical Center Lihuili Estern Hospital, Ningbo, China

**Keywords:** homoharringtonine, smad3, TGF-β, acute myeloid leukemia

## Abstract

Homoharringtonine (HHT) has long and widely been used in China for the treatment of acute myeloid leukemia (AML), the clinical therapeutic effect is significant but the working mechanism is poorly understood. The purpose of this study is to screen the possible target for HHT with virtual screening and verify the findings by cell experiments. Software including Autodock, Python, and MGL tools were used, with HHT being the ligand and proteins from PI3K-Akt pathway, Jak-stat pathway, TGF-β pathway and NK-κB pathway as the receptors. Human AML cell lines including U937, KG-1, THP-1 were cultured and used as the experiment cell lines. MTT assay was used for proliferation detection, flowcytometry was used to detect apoptosis and cell cycle arrest upon HHT functioning, western blotting was used to detect the protein level changes, viral shRNA transfection was used to suppress the expression level of the target protein candidate, and viral mRNA transfection was used for over-expression. Virtual screening revealed that smad3 from TGF-β pathway might be the candidate for HHT binding. In AML cell line U937 and KG-1, HHT can induce the Ser423/425 phosphorylation of smad3, and this phosphorylation can subsequently activate the TGF-β pathway, causing cell cycle arrest at G1 phase in U937 cells and apoptosis in KG-1 cells, knockdown of smad3 can impair the sensitivity of U937 cell to HHT, and over-expression of smad3 can re-establish the sensitivity in both cell lines. We conclude that smad3 is the probable target protein of HHT and plays an important role in the functioning mechanism of HHT.

## INTRODUCTION

Homoharringtonine (HHT) was originally extracted from the plant Cephalotaxus hainanensis, and has been used for the treatment of hematological malignancies since the 1970s [1–3] in China. Since then, this medicine has been attracting the attention of investigators worldwide and numerous investigations have been performed to examine the clinical effect [4–6], proper dosage [7, 8], drug combination^9-11^ and working mechanisms [12–16] of HHT. Recently, we have launched a national-wide, multicenter, randomized, double-blind, prospective phase III clinical trial to study the effect of an HHT-based induction regimen in de novo AML patients, the results showed that the HHT-based regimen induced higher CR ratio and longer overall survival [17]. Also, HHT plays an important role in the treatment of chronic myeloid leukemia (CML), both before and after the clinical use of tyrosine kinase inhibitors(TKIs) [6, 18–22]. And in 2010, FAD approved the use of HHT in relapsed/refractory CML. Along with the clinical investigations is the research concerning the working mechanisms of HHT, different mechanisms have been revealed, including binding with the small subunit of ribosome and interfering with the process of translation thus inhibiting protein synthesis [14], altering apoptosis-related proteins and inducing cell apoptosis [23], influencing signaling pathways, like Jak-stat5 pathway by regulating protein tyrosine kinase phosphorylation [11, 24], and inhibiting Akt pathway [13]. With all the complicated cross-talk amongst these pathways and proteins, we try to find out the initial target and pathway responsible for HHT functioning.

Virtual screening is an efficient method in novel drug discovery [25], it uses software to investigate the relationship between the ligand and the target. Protein docking is an important and effective branch of virtual screening, it simulates the interaction between a ligand and a receptor, then calculate the binding energy of the virtual conformation, and screen the candidates according to the calculated results [26].

In this study, we used HHT as the ligand, and proteins from the previously reported HHT-related pathways as candidate receptors, and screened these candidates with docking models. We have managed to find out the candidate with the greatest possibility, and verified this in AML cell lines.

## RESULTS

### Ligand-receptor docking model revealed smad3 as the best candidate for HHT binding

**Figure 1:**
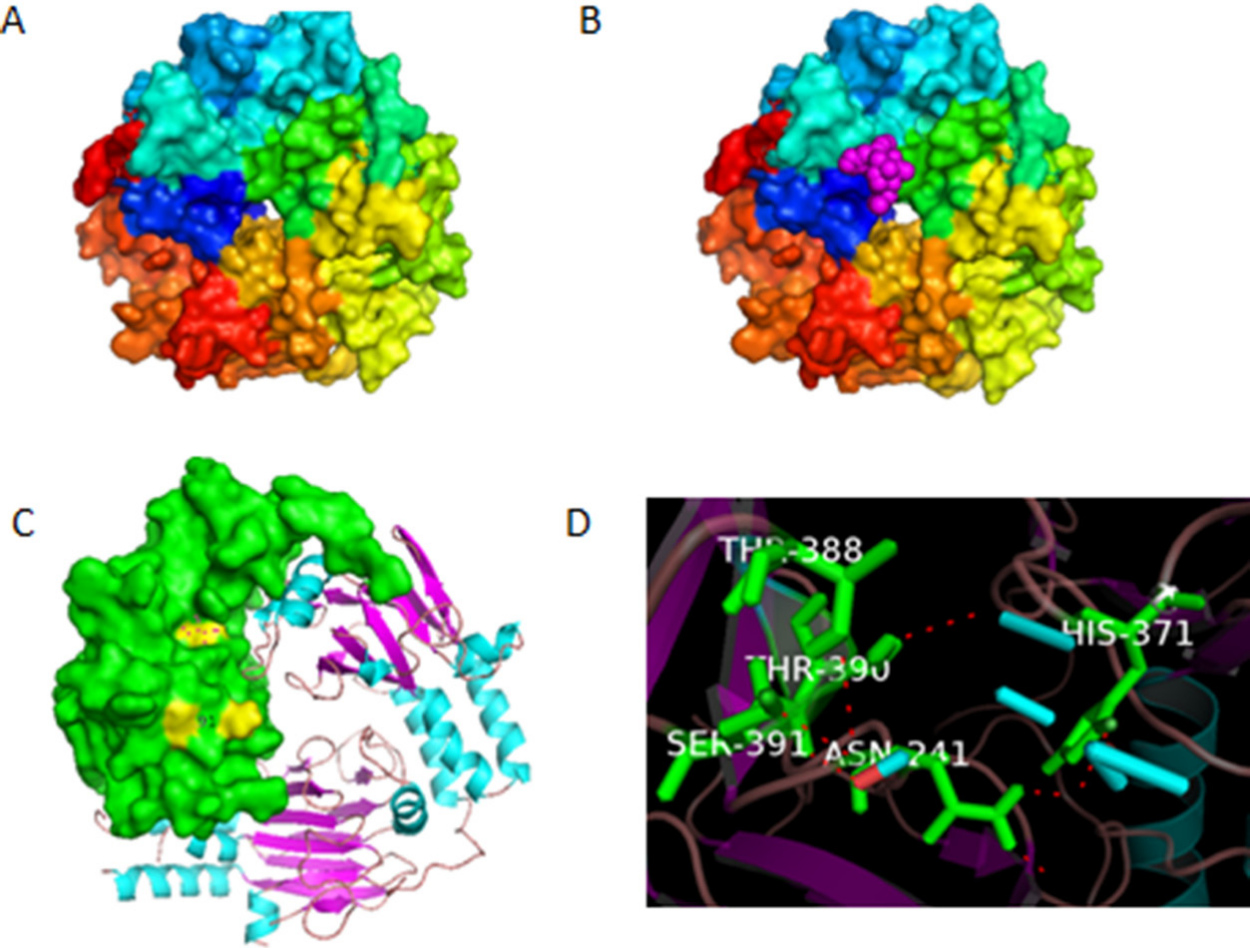
(**A**) Conformation of smad3/4 complex without homoharringtonine (HHT). (**B**) Docked conformation of smad3/4 with HHT, the ligand HHT is indicated by the pink molecule. (**C**) The estimated residues that bind with HHT, shown in yellow color, analyzed by Pymol. (**D**) Name of the residues for binding.

Present discovered mechanisms of HHT, including protein-synthesis inhibition, apoptosis induction, Jak-stat5 and Akt pathway inhibition, can only explain part of the biological effects of this dramatic medicine. To find out the target protein and the corresponding pathway responsible for the functioning mechanisms of HHT, we set the proteins from the above pathways as receptors, and dock them with HHT. These proteins include PTEN, PI3K, α-actin, androgen receptor, APPL1, Brk, cdc25A, GRB10, Hsp27, p21Cip1, PP2CA, Raf1, Smad3/4, Jak3, STAT3, Akt, smad2, smad4, TGFbR1, TGFbR2m, smad1, CYLD, Erk, mTOR, NF-kappaB, PDK1, SHIP1, SHIP2, TRAF6,etc. The docking software used in the experiment was Autodock 4.2. The software listed the docking results for each receptor as 10 conformations with the lowest free energy, within these 10 conformations, we take the one with the lowest energy as the final docking result (Table 1). From the result, we can tell that smad3/4 complex has the lowest binding energy (−8.58 kcal/mol), it could form the most stable structure with HHT, and thus has the largest probability to be the target protein. The conformation of this complex with and without HHT is shown in Figure 1A, 1B. Further analysis showed that in the binding conformation, HHT binds to the MH2 region of smad3 at the residues Asn241,His371, Thr388, Thr390 and Ser391(Figure 1C, 1D). Smad3 is one of the key proteins in TGF-β pathway, which is one of the most important pathways in cancer biology, the activation of this pathway can inhibit the proliferation of tumor cells and influence the transcription of over 500 genes [27–29]. If HHT could influence the activity of smad3 and the subsequent TGF-β pathway, it would be a reasonable explanation for the anti-leukemic activity of HHT. We took smad3 as a reasonable potential target for HHT binding.

**Table 1:** Proteins selected as receptors for docking, their PDB code and the corresponding binding energy

Protein	PDB code	Energy(kcal/mol)	Protein	PDB code	Energy(kcal/mol)
Actin	2R0O	−6.06	Akt	3MVH	−5.8
Androgen receptor	2PIV	−4..49	Ape1	4LND	−6.87
cdc25a	1C25	−5.59	GRB10	3HK0	−6.2
Hsp27	4MJH	−5.87	Jak3	4HVD	−5.4
p21	1AXC	−5.8	PDK1	4RRV	−6.36
PI3K	4KZC	−7.7	pp2CA	4N0G	−4.66
PTEN	1D5R	−4.96	raf1	3OMV	−6.02
smad1	1KHU	−6.93	Smad2	1U7V	−5.71
smad3/4	1U7F	−8.58	Smad4	1DD1	−7.97
Stat3	3CWG	−6.62	Erk	3W55	−6.13
TGFbR1	3FAA	−5.96	TGFbR2	1M9Z	−4.6
NF−kappaB	1IKN	−6.01	mTOR	2GAQ	−4.72
SHIP2	2K4P	−5.02	CYLD	2VHF	−6.16
SHIP1	2VSX	−4.5	TRAF6	3HCS	−6.44

### Homoharringtonine can activate smad3 and TGF-β pathway in AML cell lines

**Figure 2:**
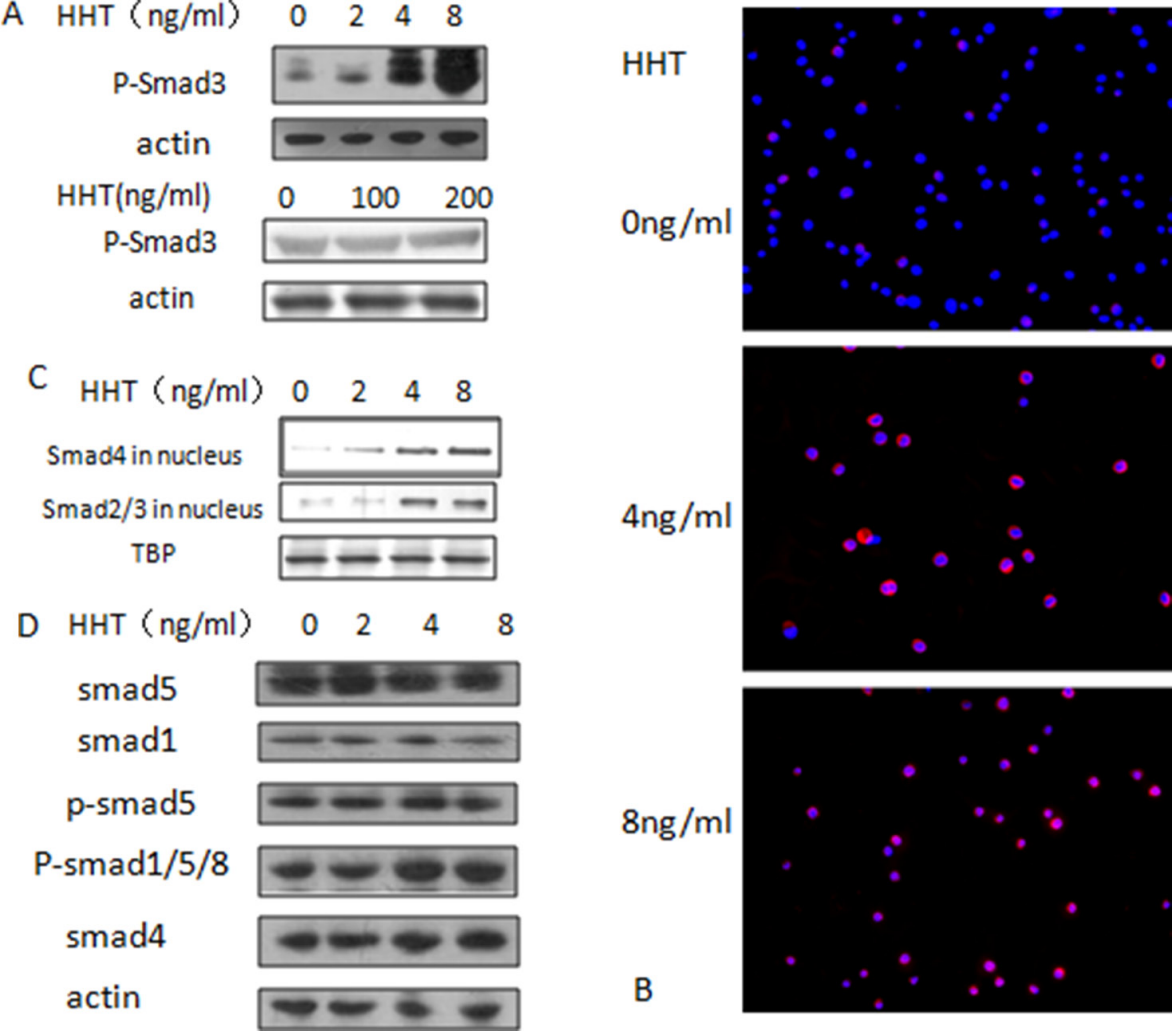
Activation of TGF-β pathway by HHT (**A**) Change of phosphorylated-smad3 at Ser425/427 in U937 cells (upper) and KG-1 cells (lower) upon concentration gradient of HHT; (**B**) Translocation of smad4, red fluorescence indicates smad4, blue fluorescence stands for nucleus stained by DAPI. (**C**) Protein level of smad2/3 and smad4 in nucleus. (**D**) Change of smad1/5/8 upon HHT treatment.

In order to verify the result of virtual screening, we try to find out whether HHT could influence the bioactivity of smad3. Since smad3 exerts its function in the TGF-β pathway through phosphorylation, conformation analysis of the docking result showed that HHT could bind with smad3 at the MH2 domain, we hypothesized that HHT might lead to the Ser423/425 phosphorylation of smad3 which in turn leads to the activation of TGF-β pathway and inhibit cell growth. To verify this hypothesis, AML cell line U937, KG-1 and THP-1 cells were treated with different concentrations of HHT for 24 hours and cell proliferation was measured by MTT assay, the IC50 value of these cell lines were: U937, 8.02 ng/ml; THP-1, 35.5 ng/ml; KG-1, 103.3 ng/ml (Supplementary Figure 1A), and these IC50 values are consistent with the smad3 protein levels of the cell lines (Supplementary Figure 1B). U937 and KG-1 were selected as the cell lines for verification because of their different sensitivities to HHT. Next we treated the U937 cells with HHT at the concentration of 0, 2, 4, 8 ng/ml for 24 h and KG-1 at 0, 100, 200 ng/ml for 24 h, then detected the Ser423/425 phosphorylation level of smad3, western blotting showed that with the increase of HHT concentration, the level of Ser423/425 phophorylated-smad3 (p-smad3) increased significantly in U937 cells, while very mild increase was observed in KG-1 cells, suggesting that HHT can induce smad3 phosphorylation in both cell lines, and this activation is consistent with their sensitivity to HHT (Figure 2A). To determine whether this phosphorylation has biological effects, smad4 translocation was detected by immunofluorescence assay, U937 cells were treated with different concentrations of HHT (0, 2, 4, 8 ng/ml) for 24 hours and the intracellular location of smad4 was detected. The result showed that, in the control group, smad4 scattered within the cell, forming a sand-like red fluorescent pattern, in the HHT 4 ng/ml group, smad4 started to gather around the nucleus and formed a ring-like pattern, while in the 8 ng/ml group, smad4 protein had entered into the nucleus, forming a disk-like pattern, indicating that upon HHT activation, smad4 protein would trans-locate from the cytoplasm into the nucleus of U937 cells (Figure 2B), this result was further verified by western-blotting of smad4 and smad2/3 in the nuclear proteins (Figure 2C), which showed that the nuclear smad4 and smad2/3 level gradually increased as the HHT concentration increased. Meanwhile, no significant change of smad1/5/8 was noticed (Figure 2D), suggesting that HHT can activate the smad2/3/4 branch but not the smad1/5/8 –BMP branch of TGF-β signaling pathway.

### The TGF-β pathway is activated over homoharringtonine functioning

**Figure 3:**
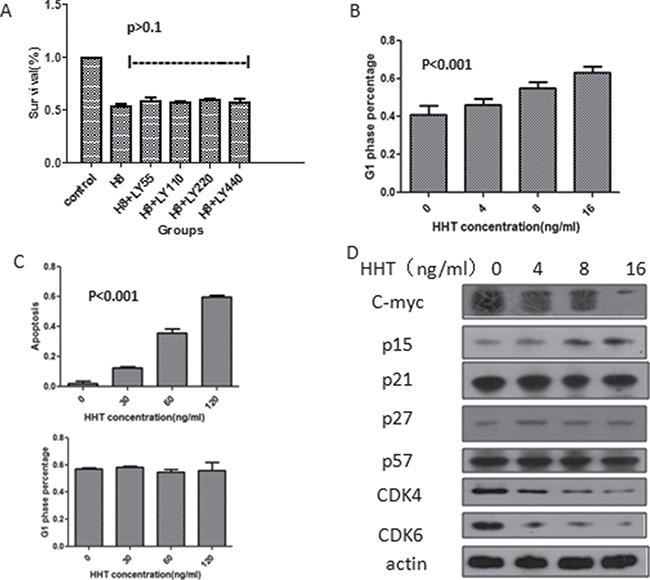
Influence of activation of TGF-β pathway on AML cell line survival (**A**) Survival of U937 cells after been treated by HHT and TbRI /TbRII inhibitor LY2109761 for 24 h (H8 means HHT 8 ng/ml, LY55 means LY2109761 55 nM, LY110 means LY2109761 110 nM, LY220 means LY2109761 220 nM, LY440 means LY2109761 440 nM). (**B**) G1 phase cell percentage of U937 cells measured by flowcytometry after treated by different concentrations of HHT, cells stained by Annexin V/PI. (**C**) Apoptosis (upper) and G1 phase cell percentage (lower) of KG-1 cells following HHT treatment at different concentrations for 24 h, detected by flowcytometry. (**D**) Protein level change of the downstream molecules following HHT treatment for 24 h at different concentrations.

We have found out that HHT can induce phosphorylation of smad3 and subsequently cause translocation of smad4, but is TGF-β pathway fully activated to initiate the downstream effects? Prior to that, we need to make sure that the activation of TGF-β pathway is due to the phosphorylation of smad3, not that of the upstream TGF-β receptors (TbRII and TbRI), TbRII and TbRI inhibitor LY2109761 was added to U937 cells to inhibit the function of these receptors, at the same time, HHT was also added, after 24 h incubation, cell proliferation was detected by MTT assay. Results showed that HHT can inhibit the proliferation of cells with or without the inhibitor, there was no significant difference (*p* > 0.1, Figure 3A), indicating that the activation of smad3 by HHT is not closely associated with the upstream TGF-β receptor activation.

To determine which cell death pathway was induced by HHT, U937 cells were treated with different concentrations (0, 4, 8, 16 ng/ml) of HHT for 24 h, cell apoptosis and cell cycle status were accessed by flowcytometry, the results showed that cell apoptosis rate were 0.14%, 0.13%, 0.14%, 0.19% respectively, and there was no significant difference amongst the experiment groups (*p* > 0.05) (Supplementary Figure 2A). Instead, HHT can arrest U937 cells at G1 phase, as the concentration increased, G1-phase cell proportion gradually increased from 40.8% without HHT, 46.2% with 4 ng/ml HHT, 55.0% with 8ng/ml HHT to 63.4% with 16 ng/ml HHT, statistical analysis showed significant difference(*p* < 0.001) (Figure 3B). Since TGF-β activation can lead to G1 phase arrest by suppressing c-myc, p15, p21 and up-regulating p27, p57. To further investigate the change of these proteins after HHT functioning, U937 cells were incubated with different concentrations of HHT for 24h, and the cells were then tested for the level of the above proteins by western blot. The result revealed that HHT could induce decrease in the protein levels of c-myc, CDK4, CDK6; increase in p15; no significant change in p21,p27 and p57 (Figure 3D), these results were all consistent with the mechanism by which TGF-β activation arrests the cell cycle, indicating that HHT could arrest the cell cycle of U937 at G1 phase by activating TGF-β pathway, inhibiting the downstream c-myc, CDK4, CDK6 and increasing p15 expression.

For KG-1 cells, the results were quite different, after incubating with HHT for 24h at different concentrations (0, 30, 60, 120 ng/ml), flowcytometry showed that there was no significant change in cell G1 phase arrest (Figure 3C, Supplementary Figure 2B), but significant increase in cell apoptosis was observed, as the concentration of HHT increased from 0, 30, 60 ng/ml to 120 ng/ml, the apoptotic cell proportion increased from 1.5%, 12.7%, 35.8% to 59.5% respectively, and statistical analysis revealed *P* < 0.001(Figure 3C), indicating that HHT can inhibit KG-1 proliferation by apoptosis induction, not cell cycle arrest.

The above results showed that HHT can inhibit the proliferation of both U937 and KG-1 cells, yet with completely different mechanisms, the former by cell cycle arrest, not apoptosis; and the latter by apoptosis induction, not cell cycle arrest. We believe this is consistent with the heterogeneity of the biological effects upon TGF-β pathway activation.

### The effects of homoharringtonine is impaired by smad3 repression

**Figure 4:**
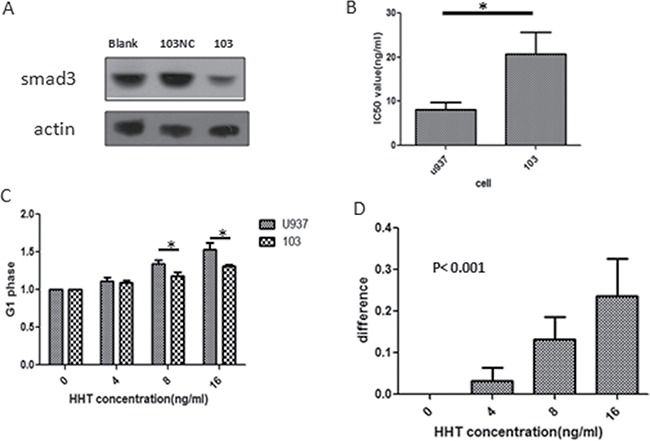
Effects of smad3-shRNA transfection on cell behavior (**A**) Change of protein level of smad3 after transfection. (103NC means U937 cells transfected with empty vectors, 103 means U937 cells transfected with smad3-shRNA) (**B**) Change of IC50 of HHT on cells before (U937, left lane) and after (103, right lane) transfection. (**C**) G1 phase percentage of U937 and 103 cells under different concentrations of HHT. (**D**) Decreasement of cell G1 phase percentage after transfection at 24 h. The y axis represents G1 phase percentage of U937 cells minus that of 103 cells under the given HHT concentrations.

In order to further investigate the importance of smad3 in HHT functioning, we artificially down-regulated the protein level of smad3 in U937 cells by lentivirus transfection, U937 cell was transfected with smad3-shRNA-loaded virus and empty vectors (smad3-shRNA-transfected U937 cells, referred to as 103 cells; empty vector transfected U937 cells, referred to as 103NC cells. smad3 shRNA sequences are shown in Supplementary Table 1), smad3 protein level was detected to have been significantly lowered by western blot (Figure 4A). U937 cells and 103 cells were treated with different concentrations of HHT for 24h and their proliferation were measured by MTT assay, the result showed that after smad3 shRNA transfection, compared with U937 cells, the IC50 value of 103 cell has increased from 8.02 ng/ml to 20.79 ng/ml (*p* < 0.05) (Figure 4B), indicating that after the knockdown of smad3, U937 cell became less sensitive to HHT.

Since HHT could inhibit U937 proliferation by G1 phase arrest, we next examined the effect of smad3 knockdown on this, U937 and 103 cell were treated with the same HHT concentration gradient (0, 4, 8.16 ng/ml) for 24 h, then their cell cycle status were assessed by flowcytometry, the result showed that G1 phase arrest became significantly less following smad3 knockdown, in U937 cells, G1 phase cell percentage following the above concentration gradient of HHT at 24 h were 40.8%,46.2%,55% and 63.4%, while that of 103 cell were 42.3%,48.7%,49.2% and 54.7%, statistical analysis showed that the G1 phase arrest for 103 cell is significantly less than that of U937 cells at the concentrations of 8 ng/ml and 16 ng/ml (*p* < 0.001) (Figure 4C and 4D). These results suggest that knockdown of smad3 in U937 cell could impair its sensitivity to HHT by attenuating G1 phase arrest.

### Smad3 re-expression and over-expression increase the sensitivity of AML cell lines to HHT

**Figure 5:**
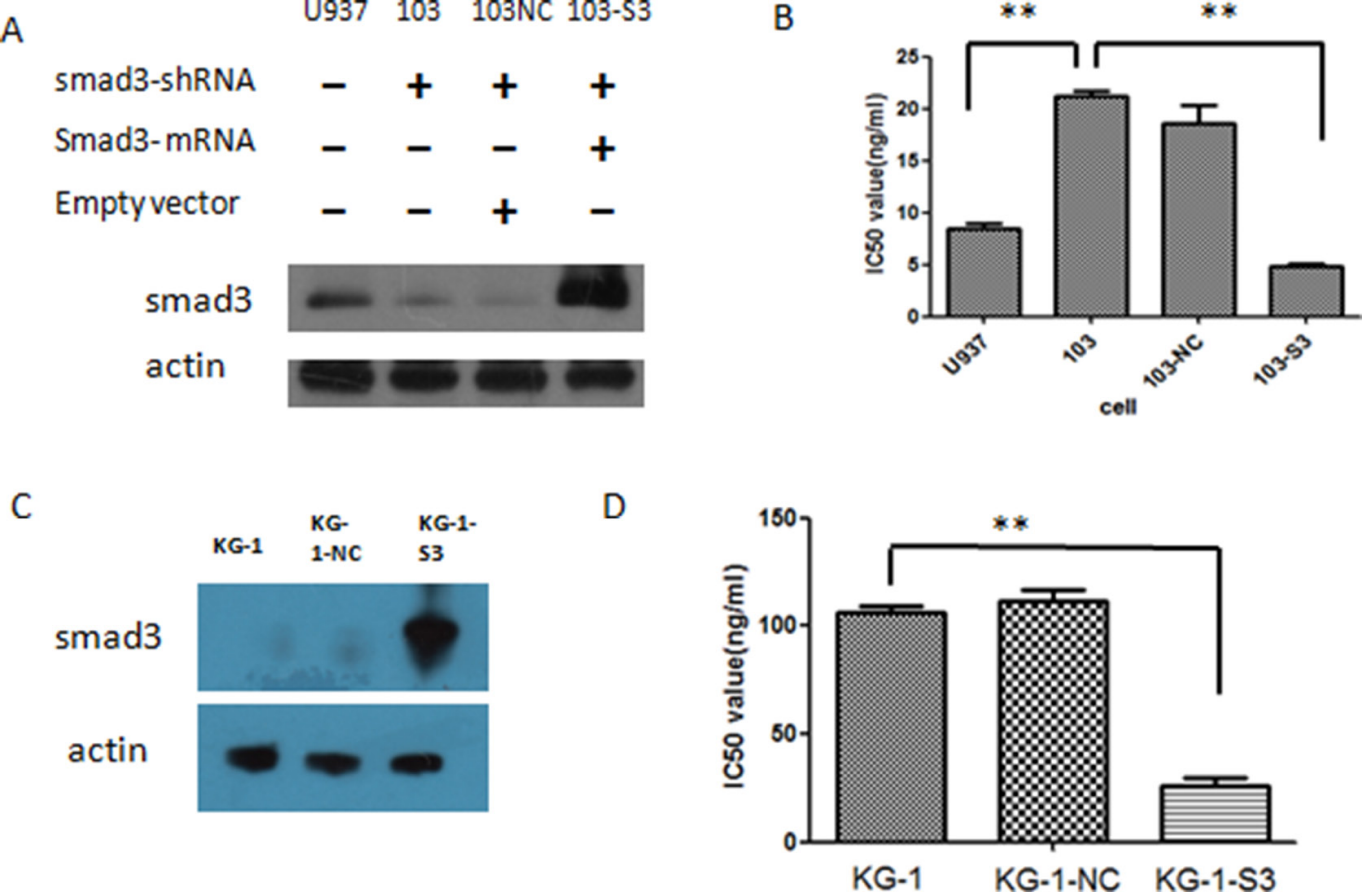
Effects of smad3 re-expression and over-expression in AML cell lines (**A**) Level of smad3 after knock-down and re-expression in U937 cells by western blot. (**B**) IC50 values of the cells with and without transfection. 103 refers to U937 cells transfected with smad3 shRNA, 103NC refers to 103 cells transfected with empty vectors, 103-S3 refers to 103 cells transfected with smad3 mRNA. (**C**) Protein level of smad3 in KG-1 cells following smad3 over-expression. KG-1-NC refers to KG-1 cells transfected with empty vectors, KG-1-s3 refers to KG-1 cells transfected with smad3-mRNA vectors. (**D**) IC50 values of KG-1 cells before and after smad3 mRNA transfection.

Since we have found out that knockdown of smad3 could increase the IC50 value of U937 to HHT, next we try to find out whether that sensitivity impairment could be rescued by smad3 re-expression or be increased by smad3 over-expression. Smad3 mRNA was transfected to 103 cells by retro-virus, and the smad3 level of U937, 103, 103NC and smad3-reexpression-103 cells (referred to as 103-S3 cells) was evaluated by western blot, the result showed that 103 cells has significantly lower level of smad3 as described previously, and103-S3 cells had significantly higher level of smad3, even higher than that of the original U937 cells (Figure 5A), indicating that the re-expression was successful. Then we treated these cells with different concentrations of HHT for 24 h, and cell proliferation was then assessed by MTT assay, the result showed that the IC50 value for 103-S3 cells was 4.89 ng/ml, which was significantly less than that of 103 cells (*p* < 0.05) (Figure 5B). Interestingly, we also found that the 103-S3 cells were the most sensitive to HHT since they have the highest level of smad3 protein, even more sensitive than the original U937 cells, indicating that the re-expression of smad3 in the low-smad3-expressing 103 cells could re-establish their sensitivity to HHT, and also, the sensitivity of these cells to HHT was consistent with their smad3 protein levels.

Since KG-1 cells had a relatively low expression level of smad3 and were insensitive to HHT, we wonder whether over-expression of smad3 in KG-1 cells could improve the sensitivity. The same smad3 mRNA was transfected to KG-1 cells as well as empty vectors, and smad3 protein level verified by western blot, the result showed that KG-1-NC (KG-1 cells with empty vector) had the similar level of smad3 as KG-1, while KG-1-S3 (KG-1 cells transfected with smad3 mRNA) had a significantly higher level of smad3 (Figure 5C). These cells were then incubated with different concentrations of HHT for 24 h, MTT assay was performed to access the proliferation, and their IC50 value calculated as previously described, the result showed that the IC50 value for KG-1, KG-1-NC and KG-1-S3 were 106.4 ng/ml, 111.4 ng/ml and 26.3 ng/ml, respectively, statistics analysis showed significant difference between the IC50 values of KG-1 and KG-1-S3 (Figure 5D), indicating that over-expression of smad3 could improve the sensitivity of KG-1 toward HHT.

## DISCUSSION

In our experiment, we found by docking that HHT may bind with the MH2 domain of smad3 at the residues Asn241, His371, Thr388, Thr390 and Ser391, and can induce the phosphorylation of Ser423/425 of smad3, how the phosphorylation is induced is not yet clear, conformational change of smad3 and exposure of phosphorylation site may be an possible mechanism, we will look into the exact mechanism in our future study.

One striking finding of this study is that HHT could activate the TGF-β pathway, one of the most important pathways of epithelium-derived carcinoma, that is, an effective medicine for hematological malignancies could activate a very important pathway in solid carcinomas. Several immediate questions would be: What are the roles of TGF-β pathway in the pathogenesis and progression of hematologic malignancies? Also, in epithelial carcinoma, activation of TGF-β pathway can inhibit carcinogenesis but promote metastasis [29, 30], this paradox has made the clinical use of TGF-β-pathway-related drugs highly debatable. Since HHT can activate TGF-β pathway in AML cells, what would be its performance toward epithelial carcinomas? What would be HHT's performance toward this paradox? AML and CML do not involve metastasis, would TGF-β pathway activators inhibit the proliferation of these cells? Would these activators be another option for the treatment of these hematological malignancies? Our study abridges hematological malignancies and epithelium-derived malignancies by HHT and TGF-β pathway.

We could only conclude that HHT can activate the TGF-β pathway, probably by directly acting on smad3 since we could not provide evidence for the direct binding between HHT and smad3. Originally, we tried to link a tag, like biotin, to the side chain of HHT but failed, because HHT is pharmacologically very steady, and tagging it with biotin is nearly impossible without altering the properties of HHT. Another concern is, HHT is a small molecule, if we tag it with a protein mark in order to detect its target using immune-precipitation, the volume of the protein-tagged HHT might be much larger than the conformational space at the target protein, and thus generate false positive results, we will try isotope labeling in our future study.

In summary, we have found out and proved that samd3 from TGF-β pathway is a probable target for HHT, and through phosphorylating smad3 in the AML cell lines, HHT could active the TGF-β pathway, which in turn arrest the cell cycle at G1 phase or induce cell apoptosis depending on the background, and thus inhibit the proliferation of these cells. Further, the activation of TGF-β pathway is important for the function of HHT, knockdown of smad3 could impair the inhibitory function and re-expression of smad3 could rescue it, over-expression of smad3 in an originally insensitive AML cell line could improve its sensitively to HHT. We provided an explanation for the clinical effectiveness of HHT in AML patients. Also, our methodology of employing docking in target protein screening has proven to be effective, we believe this method could also be used in the target screening for other small molecules.

## MATERIALS AND METHODS

### Software and resources

Software used in this study include: Python 2.5 from www.python.org, MGL tools1.5.4 from http://mgltools.scripps.edu/, Autodock 4.2 from http://autodock.scripps.edu/. These programs were downloaded and installed following instructions from their websites.

The structure of homoharringtonine was obtained from Pubchem, then processed with PRGDRG program from http://davapc1.bioch.dundee.ac.uk/cgi-bin/prodrg, we got the names of proteins which serve as receptors for docking from previous literature, and their 3D structures were downloaded from PDB (protein data bank, http://www.rcsb.org/pdb/home/home.do).

### Cell culture

Human acute myeloid leukemia cell line U937, KG-1, THP-1 were purchased from Institute of Biochemistry and Cell Biology, Chinese Academy of Sciences, Shanghai, and were cultured as previously described^13^.

### Chemicals and antibodies

Antibodies: smad3, p-smad3, smad2, smad4, smad1, smad5, smad8, p15, p21, p27, p57, c-myc, CDK4, CDK6, β-actin, TBP antibodies were purchased from Cell Signaling Technology. Goat anti-mouse antibody, goat anti-rabbit antibody were also from Cell Signaling Technology. TbRI and TbRII inhibitor LY2109761 was purchased from Selleckchem. Homoharringtonine was purchased from Sigma.

### Cell proliferative assay

Cell proliferation was detected by MTT assay (Sigma), cell apoptosis was detected by flowcytometry using Annexin V/ PI staining(Roche company). Cell cycle assay was performed by flowcytometry using PI staining as previously described [13].

### Immunofluorescence test

Cells were treated with different concentrations of HHT, then collected and fixed with 4% paraform solution. Carefully smear the cell solution on a glass plate, wash the cells with 0.5% tristone solution, use goat serum (Gibico) to block nonspecific binding site, incubate the cells with anti-smad4 antibody for 12 h, then incubate with fluorescence-labeled secondary antibody, and observe the result under a fluorescence microscope (Olympus).

### Transfection

Smad3 shRNA was synthesized and packaged into retrovirus by Shanghai Genechem Co. Cells were transfected with virus according to the protocol provided by the producer.

### Statistical analysis

Results were analyzed using SPSS 16.0, *p* < 0.05 was noted with *, and *p* < 0.01 was noted with **.

## SUPPLEMENTARY MATERIALS FIGURES AND TABLES


