# Supplementary material for: Homoharringtonine targets Smad3 and TGF-β pathway to inhibit the proliferation of acute myeloid leukemia cells
Source: Oncotarget. 2017 Apr 8;8(25):40318–26. doi: 10.18632/oncotarget.16956 (PMC5522237; doi:10.18632/oncotarget.16956)
Supplement: Supplementary file 1 [file oncotarget-08-40318-s001.pdf]

Homoharringtonine targets Smad3 and TGF-β pathway to inhibit the proliferation of acute myeloid leukemia cells

Supplementary Materials

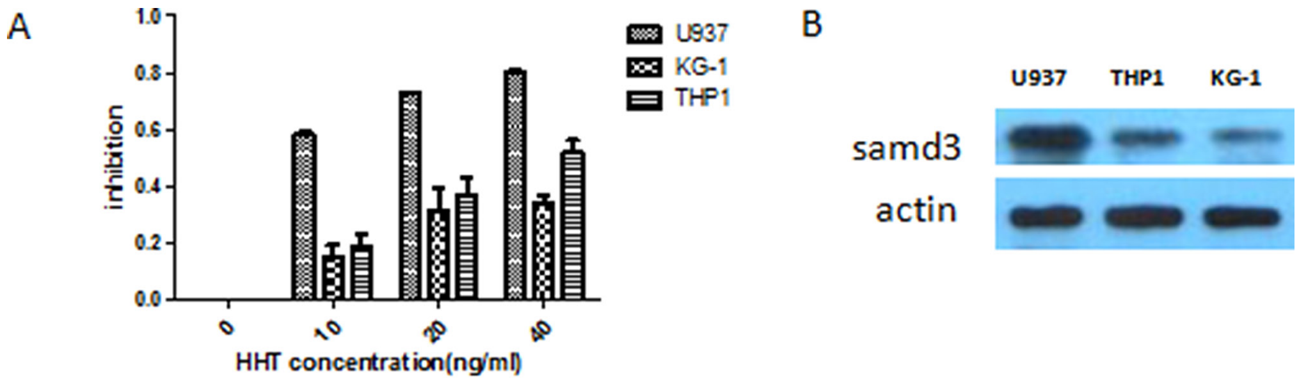

Supplementary Figure 1: (**A**) Effects of homoharringtonine on the proliferation of AML cell line U937, THP1 and KG-1, y axis indicates the inhibition ratio. (**B**) Protein level of smad3 in different AML cell lines by western blot.

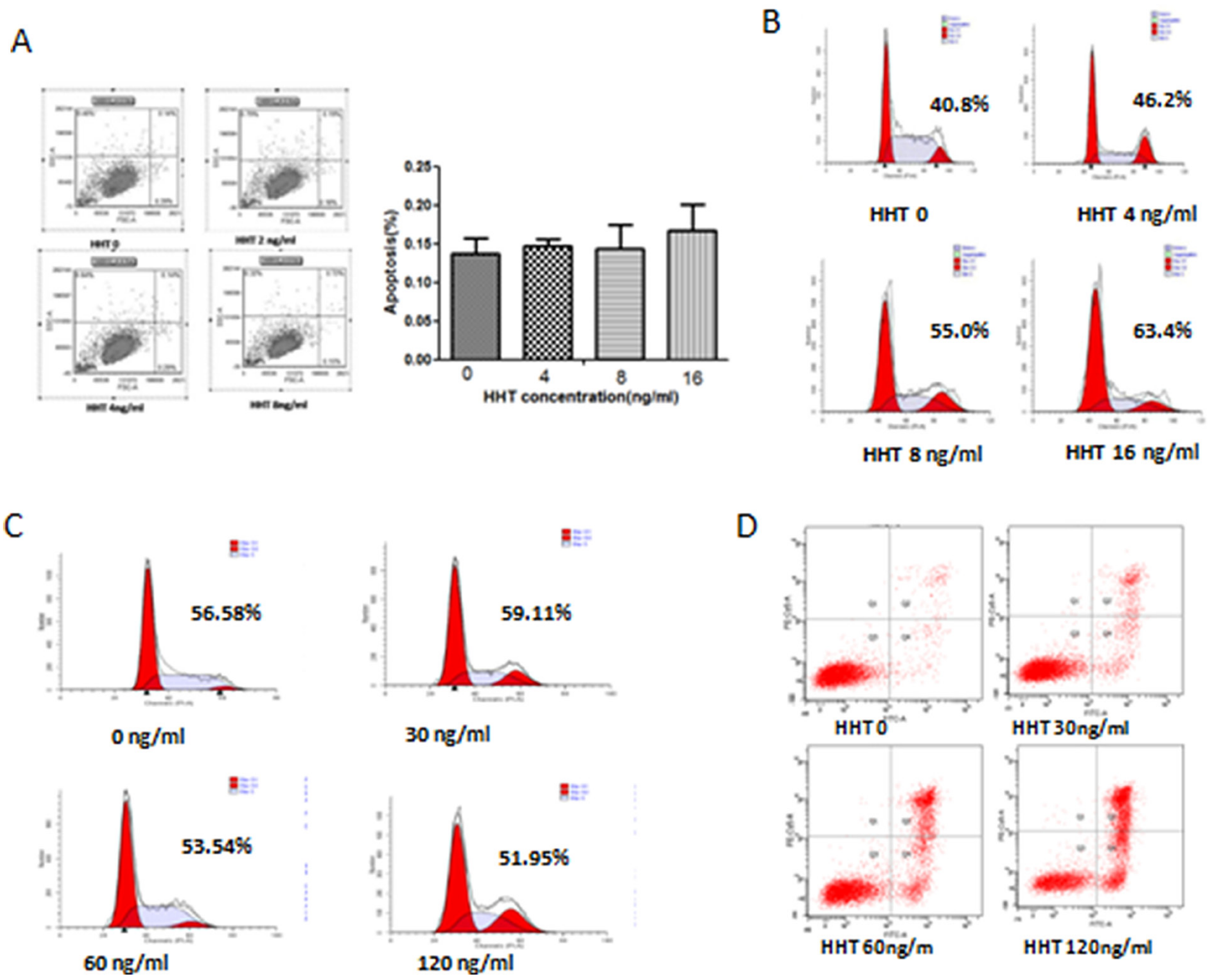

Supplementary Figure 2: Effects of homoharringtonine on cell cycle and apoptosis of AML cell lines. (**A**) Apoptosis of U937 induced by different concentrations of HHT, accessed with flowcytometry. (**B**) G1 phase percentage of U937 cells treated with different concentrations of HHT, accessed with flowcytometry. (**C**) G1 phase percentage of KG-1 cells treated with different concentrations of HHT. (**D**) Apoptosis of KG-1 cells treated with different concentrations of HHT.

Supplementary Table 1: Sequences of smad3 shRNA used for transfection

NO.	5′	STEM	Loop	STEM	3′
SMAD3-RNAi(27102)-a	Ccgg	ccGCTGTTCCAGTGTGTCTTA	CTCGAG	TAAGACACACTGGAACAGCGG	TTTTTg
SMAD3-RNAi(27102)-b	aattcaaaaa	ccGCTGTTCCAGTGTGTCTTA	CTCGAG	TAAGACACACTGGAACAGCGG	
SMAD3-RNAi(27103)-a	Ccgg	caTCTCCTACTACGAGCTGAA	CTCGAG	TTCAGCTCGTAGTAGGAGATG	TTTTTg
SMAD3-RNAi(27103)-b	aattcaaaaa	caTCTCCTACTACGAGCTGAA	CTCGAG	TTCAGCTCGTAGTAGGAGATG	
SMAD3-RNAi(27104)-a	Ccgg	ccCAGCACATAATAACTTGGA	CTCGAG	TCCAAGTTATTATGTGCTGGG	TTTTTg
SMAD3-RNAi(27104)-b	aattcaaaaa	ccCAGCACATAATAACTTGGA	CTCGAG	TCCAAGTTATTATGTGCTGGG	

27103 was used in the experiment according to efficacy.
